# A machine learning model accurately identifies glycogen storage disease Ia patients based on plasma acylcarnitine profiles

**DOI:** 10.1186/s13023-025-03537-2

**Published:** 2025-01-09

**Authors:** Joost Groen, Bas M. de Haan, Ruben J. Overduin, Andrea B. Haijer-Schreuder, Terry GJ Derks, M. Rebecca Heiner-Fokkema

**Affiliations:** 1 Laboratory of Metabolic Diseases, Department of Laboratory Medicine, University Medical Center Groningen, University of Groningen, Hanzeplein 1, Postbus, Groningen, 30001 - 9700 RB the Netherlands; 2 Laboratory of Special Chemistry, Department of Laboratory Medicine, University Medical Center Groningen, University of Groningen, Groningen, 9700 RB The Netherlands; 3 Division of Metabolic Diseases, Beatrix Children’s Hospital, University Medical Center Groningen, University of Groningen, Groningen, 9700 RB The Netherlands

**Keywords:** Rare diseases, Machine learning, Inborn metabolic diseases, Artificial intelligence, Glycogen storage disease, Acylcarnitines

## Abstract

**Background:**

Glycogen storage disease (GSD) Ia is an ultra-rare inherited disorder of carbohydrate metabolism. Patients often present in the first months of life with fasting hypoketotic hypoglycemia and hepatomegaly. The diagnosis of GSD Ia relies on a combination of different biomarkers, mostly routine clinical chemical markers and subsequent genetic confirmation. However, a specific and reliable biomarker is lacking. As GSD Ia patients demonstrate altered lipid metabolism and mitochondrial fatty acid oxidation, we built a machine learning model to identify GSD Ia patients based on plasma acylcarnitine profiles.

**Methods:**

We collected plasma acylcarnitine profiles from 3958 patients, of whom 31 have GSD Ia. Synthetic samples were generated to address the problem of class imbalance in the dataset. We built several machine learning models based on gradient-boosted trees. Our approach included hyperparameter tuning and feature selection and generalization was checked using both nested cross-validation and a held-out test set.

**Results:**

The binary classifier was able to correctly identify 5/6 GSD Ia patients in a held-out test set without generating significant amounts of false positive results. The best model showed excellent performance with a mean received operator curve (ROC) AUC of 0.955 and precision-recall (PR) curve AUC of 0.674 in nested CV.

**Conclusions:**

This study demonstrates an innovative approach to applying machine learning to ultra-rare diseases by accurately identifying GSD Ia patients based on plasma free carnitine and acylcarnitine concentrations, leveraging subtle acylcarnitine abnormalities. Acylcarnitine features that were strong predictors for GSD Ia include C16-carnitine, C14OH-carnitine, total carnitine and acetylcarnitine. The model demonstrated high sensitivity and specificity, with selected parameters that were not only robust but also highly interpretable. Our approach offers potential prospect for the inclusion of GSD Ia in newborn screening. Rare diseases are underrepresented in machine learning studies and this work highlights the potential for these techniques, even in ultra-rare diseases such as GSD Ia.

**Supplementary Information:**

The online version contains supplementary material available at 10.1186/s13023-025-03537-2.

## Background

Glycogen storage disease (GSD) Ia, also called Von Gierke disease, is an ultra-rare inherited disorder of carbohydrate metabolism caused by deficiency of the enzyme glucose-6-phosphatase (G6Pase) [1]. This disease leads to an inability to generate glucose in the terminal step of glycogenolysis and gluconeogenesis. Patients typically present in the first year of life with hepatomegaly and/or symptoms associated with hypoglycemia. Biochemically, these patients often have fasting (hypoketotic) hypoglycemia, hyperlipidemia, hypertriglyceridemia, hyperuricemia and hyperlactatemia [2]. 

GSD Ia patients also exhibit altered lipid metabolism, characterized by increased lipogenesis that leads to hypertriglyceridemia [3]. In addition, high levels of malonyl-CoA in these patients inhibit carnitine palmitoyltransferase I (CPT I), the rate-controlling step of mitochondrial fatty acid oxidation. G6Pase deficient mice also demonstrate altered mitochondrial morphology and impaired mitochondrial function in their livers [4]. Rossi et al. recently reported increased expressions and activities of enzymes involved in the Krebs cycle and mitochondrial fatty acid oxidation in GSD Ia patients [5]. Collectively, these findings illustrate that lipid metabolism and mitochondrial fatty acid oxidation are altered in GSD Ia patients, which could be reflected in their plasma acylcarnitine profiles.

The biochemical workup of pediatric patients with hypoglycemia almost invariably includes acylcarnitine profiling [6]. In addition, acylcarnitine profiling is used in many newborn screening programs for the diagnosis of fatty acid oxidation disorders and organic acidemias [7, 8]. Deficiencies of specific enzymes involved in mitochondrial fatty acid oxidation or branched-chain amino acid metabolism result in the build-up of specific acylcarnitine species from accumulating acyl-CoA’s [9]. For example, C8-carnitine is markedly increased in medium chain acyl-CoA dehydrogenase deficiency (MCADD), C14:1-carnitine in very long chain acyl-CoA dehydrogenase deficiency (VLCADD) and C3-carnitine in propionic acidemia or methylmalonic acidemia. Similarly, the accumulation of specific acylcarnitine species in GSD Ia could be detected.

Machine learning (ML) algorithms have proven to be powerful tools for the construction of classification models using complex biochemical data [10–14]. The application of ML models in rare diseases is complicated by very small patient populations, extremely unbalanced datasets and high data dimensionality. A literature overview of studies using ML in rare diseases found inherited metabolic diseases (IMDs) to be an underrepresented disease group [15]. This is surprising as IMDs are one of the most common groups of rare diseases. Since effective biomarkers for many IMDs are currently limited, machine learning models could enhance diagnostic testing by combining individually weaker biomarkers into a robust ‘compound biomarker’.

In this study, we constructed an ML model to identify GSD Ia patients based on their plasma acylcarnitine profiles. This study illustrates the possible application of ML in rare diseases and proposes possible solutions to address the unique challenges of rare disease datasets, including low positive sample size, imbalanced data and noise.

## Materials and methods

### Data preparation

The dataset contains lithium heparin plasma acylcarnitine profiles measured in routine clinical service in the Laboratory of Metabolic Diseases of the University Medical Center Groningen (The Netherlands) between 2005 and 2024. These profiles were measured as part of the diagnostic metabolic screening of patients suspected of an IMD, or for follow-up of known IMD patients. Note that the University Medical Center Groningen is a national center of expertise in hepatic glycogen storage diseases in the Netherlands.

Plasma acylcarnitines were analyzed underivatized using flow-injection tandem mass spectrometry, as described elsewhere [16]. During the time period of sample collection the acylcarnitine method was recalibrated twice, first in 2016, the second calibration in 2021. Concentrations before 2016 were calculated based on a minimal set of internal standards. In this period, external quality control samples became available, revealing biases for some acylcarnitines. Based on that, we introduced calibration curves, using standards for most available acylcarnitines and the same set of internal standards. In 2021, we changed to commercially available sets of standards and internal standards. Reference values were corrected for changes in the response factors. For this study, we applied these response factor changes to correct for the two recalibrations.

The initial dataset consisted of 8305 acylcarnitine profiles and consisted of 29 features. If more than 60% of data was missing, profiles were excluded (*n* = 74). At this point, repeated profiles of individual non-GSD Ia patients were removed (*n* = 4156). The final dataset contained 4075 profiles of 3958 unique patients, of which 31 are GSD Ia patients.

The non-GSD Ia patient group in the dataset is highly diverse, consisting of 326 patients with elevated total carnitine levels. This group includes individuals diagnosed with various IMDs, among whom 34 have a different type of GSD other than GSD Ia. For a detailed breakdown of the patients in this dataset, see Supplemental Table 1.

There was a single pre-diagnostic sample in the dataset of a GSD Ia patient.The total number of acylcarnitine profiles from GSD Ia patients is 148. The final dataset was divided into a training set (3272 profiles, 29 features, and profiles of 25 GSD Ia patients) and a test set (803 profiles, 29 features, and profiles of 6 GSD Ia patients).

Imputation of data was performed as quantitative testing for several acylcarnitine species (C5:1, C3DC/C4OH, C4DC/C5OH, C5DC, C12OH, C14OH, C16OH, C18:1OH, C18OH, C16DC and C18DC) was introduced during the time our data was collected. We performed K-Nearest Neighbor (KNN) imputation for missing values in the training and test set based on the training set data using sklearn’s KNNImputer. Approximately 17% of samples needed imputation of these acylcarnitine species. After this imputation step, a feature engineering step was performed to include several relevant acylcarnitine ratios used in our local clinical practice in addition to other potentially useful ratios [8]. This resulted in a total of 62 features in our final dataset.

### Statistical analysis

We used Mann-Whitney U tests to determine whether the distribution of specific acylcarnitine species was significantly different between GSD Ia patients and non-GSD Ia patients. Based on this initial analysis, novel acylcarnitine ratios that would be expected to be specific for GSD Ia were formulated.

### Machine learning procedure

The ML algorithms used in this study are random forests (RF), extreme gradient boosting (XGBoost) and CatBoost. Models were trained, tuned and tested using Python (version 3.12) and scikit learn (sklearn, version 1.3.2). The full script used for data preparation, the construction of ML models and model evaluation, including a full system requirements file with package versions, is available on GitHub [17]. 

Synthetic datapoints were generated by the oversampling of training data using Support Vector Machine Synthetic Minority Over-sampling Technique (SVMSMOTE) [18]. Undersampling was performed using the RandomUnderSampler of scikitlearn.

Predictive performance was evaluated using nested, stratified k-fold cross-validation (CV). Samples from GSD Ia patients in the training set were not deduplicated to utilize all information contained in these samples for model training. The result is that different samples of different GSD Ia patients can show up in multiple validation folds. To prevent data leakage and bias, we included the following procedures in each nested CV iteration:

- We deduplicated validation sets in the inner and outer loops to ensure that only the first sample of each individual patient was included in the validation sets.
- Samples from patients included in the validation sets were removed from the respective training sets.

We performed hyperparameter tuning and feature selection in the inner loop (k = 10). Hyperparameters were tuned using the Optuna package. Features were selected based on feature importances determined in the inner loop. Feature importances were determined using prediction value change: the impact of replacing a feature value by a random value on model predictions. The outer loop (k = 10) was used to assess performance and generalization of the model.

Nested CV performance was assessed using the area under the curves (AUC) of receiver operating characteristic (ROC) curves and Precision-Recall (PR) curves. F4-scores were also calculated. The 95% confidence intervals of the ROC and PR AUC were calculated from the performances of the outer loop folds. A final model was selected based on optimal AUC of the PR curve.

The final model with optimized hyperparameters was also used to evaluate the held-out test set to evaluate performance on out-of-sample data. We made SHapley Additive exPlanations (SHAP) summary plots and feature importance plots to assess the model. ROC-curves, PR-curves and F4 scores were used to check performance of the final model on the test set. Optimal thresholds were determined by highest F4 scores and confusion matrices at these thresholds were constructed. The 95% confidence intervals of the ROC and PR AUC and F4-scores were determined using bootstrapping with 10,000 bootstraps. Sensitivity, specificity, positive predictive value (PPV) and negative predictive value (NPV) were calculated from confusion matrices and 95% confidence intervals were determined using the Wilson score interval.

## Results

### Exploratory analysis and feature engineering

**Fig. 1:**
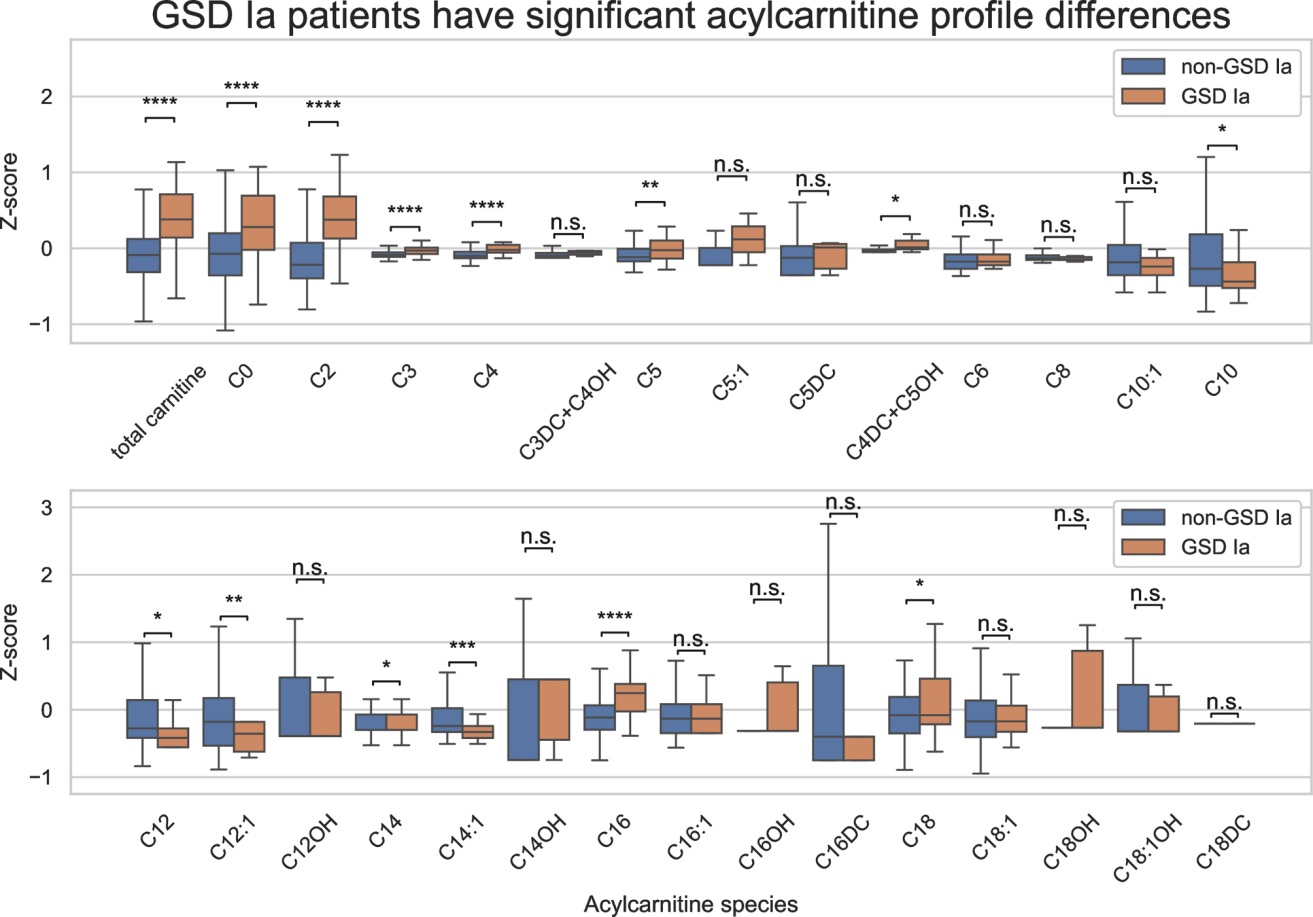
Normalized acylcarnitine distributions for GSD Ia patients and non-GSD Ia patients. Z-scores were calculated for each acylcarnitine species from the mean and standard deviations of the non-GSD Ia samples. Boxes represent the median ± interquartile range (IQR) and whiskers represent 1.5 × IQR. Outliers are not shown. The stars on the boxplot denote the levels of significance based on the results of Mann-Whitney U tests: n.s. for not significant, * for *p* < 0.05, ** for *p* < 0.01, *** for *p* < 0.001 and **** for *p* < 0.0001

We performed analyses of the dataset prior to model construction in order to determine which acylcarnitine species were significantly elevated or decreased in GSD Ia patients. The results of these analyses are provided in Fig. 1. Supplemental Figs. 1–3 show the non-normalized results.

We observed significantly elevated total and free carnitine in GSD Ia patients. Short chain acylcarnitine concentrations were significantly higher, with the exception of C3DC + C4OH, C5:1 and C5DC where there was no significant difference. Medium chain acylcarnitines showed no significant difference except lower C10, C12 and C12:1 in GSD Ia patients. Long chain acylcarnitines showed a mixed picture. C16 and C18 carnitines are elevated in GSD Ia patients and C14 and C14:1 significantly decreased.

We used this information to formulate several acylcarnitine ratios that could be used as features in a machine learning model. These include, among others, C16/(C10 + C12), C16/C14:1 and C16/(C14 + C14:1) ratios.

### Machine learning models accurately identify GSD Ia patients

The performance of Random Forest, XGBoost and CatBoost models combined with over- and/or undersampling for the identification of GSD Ia patients is summarized in Table 1.

**Table 1:** Performance metrics of different machine learning algorithms in nested, stratified k-fold CV. Mean AUC of the ROC and PR curves and F4 scores were calculated over 10 outer folds. The best performing model according to PR AUC is shown in bold. Feature selection was not performed for these models

Algorithm	Over- and/or Undersampling	Mean ROC AUC	Mean PR AUC	Mean F4 score
Random Forest	Neither	0.927 [0.907–0.946]	0.571 [0.481–0.660]	0.079 [0.000–0.178]
	Over	0.928 [0.899–0.957]	0.607 [0.514–0.701]	0.370 [0.277–0.464]
	Both	0.935 [0.903–0.966]	0.599 [0.558–0.640]	0.648 [0.561–0.736]
XGBoost	Neither	0.945 [0.933–0.958]	0.631 [0.563–0.700]	0.324 [0.219–0.429]
	Over	0.951 [0.935–0.966]	0.612 [0.521–0.704]	0.477 [0.374–0.579]
	Both	0.929 [0.911–0.947]	0.595 [0.539–0.652]	0.659 [0.609–0.709]
CatBoost	Neither	0.945 [0.897–0.968]	0.612 [0.510–0.714]	0.248 [0.113–0.382]
	**Over**	**0.950 [0.928–0.973]**	**0.648 [0.603–0.694]**	**0.416 [0.337–0.495]**
	Both	0.934 [0.909–0.958]	0.565 [0.497–0.633]	0.639 [0.598–0.679]

Generally, XGBoost and CatBoost outperformed the Random Forest algorithm. Overall, oversampling using SVMSMOTE increased performance as determined by AUC of the Precision-Recall curve. Combining random undersampling with subsequent oversampling using SVMSMOTE resulted in lower PR AUC scores but significantly higher F4 scores. This means that using both under- and oversampling increases recall but sacrifices precision.

### Feature selection

We chose to evaluate models based on the optimization of the AUC of the PR curve to find a good balance between recall and precision. The CatBoost algorithm yielded the highest PR AUC score using SVMSMOTE oversampling. Our next step was to explore feature selection. We performed feature selection in the inner loop of nested CV using CatBoost. Feature importances were calculated using Prediction Value Change (PVC) and features with importances above a certain percentile threshold were selected for further evaluation in the outer fold. Specifically, we evaluated different percentile thresholds to determine the optimal threshold for feature selection. These results are shown in Table 2.

**Table 2:** CatBoost models with different feature selection cutoffs were evaluated. The feature selection parameters yielding the best performing model are shown in bold

Cutoff percentile of feature importance	Mean ROC AUC	Mean PR AUC	Mean F4 score	Number of features selected after Nested CV
100%	0.950 [0.928–0.973]	0.648 [0.603–0.694]	0.416 [0.337–0.495]	64
90%	0.951 [0.931–0.972]	0.661 [0.597–0.726]	0.481 [0.396–0.567]	64
80%	0.949 [0.927–0.970]	0.672 [0.604–0.741]	0.476 [0.395–0.558]	62
70%	**0.955 [0.934–0.975]**	**0.674 [0.604–0.743]**	**0.488 [0.423–0.552]**	**51**
60%	0.948 [0.920–0.975]	0.663 [0.596–0.731]	0.466 [0.395–0.538]	43
50%	0.953 [0.928–0.978]	0.674 [0.601–0.746]	0.488 [0.404–0.573]	37
40%	0.957 [0.934–0.981]	0.666 [0.580–0.752]	0.462 [0.382–0.542]	28
30%	0.953 [0.934–0.972]	0.624 [0.538–0.710]	0.390 [0.276–0.505]	18
20%	0.951 [0.934–0.968]	0.637 [0.584–0.691]	0.420 [0.313–0.528]	10
10%	0.922 [0.897–0.946]	0.579 [0.494–0.663]	0.430 [0.321–0.539]	6

Interestingly, feature selection benefited model performance to a degree. Selecting the top 70% of feature importances (51 features) yielded the best performance, with an PR AUC of 0.674. We found that even when selecting only 6 features (the top 10% of features by feature importance scores), our model still performed quite well. Supplemental Fig. 4 shows the performance of this simple model and the selected features.

**Fig. 2:**
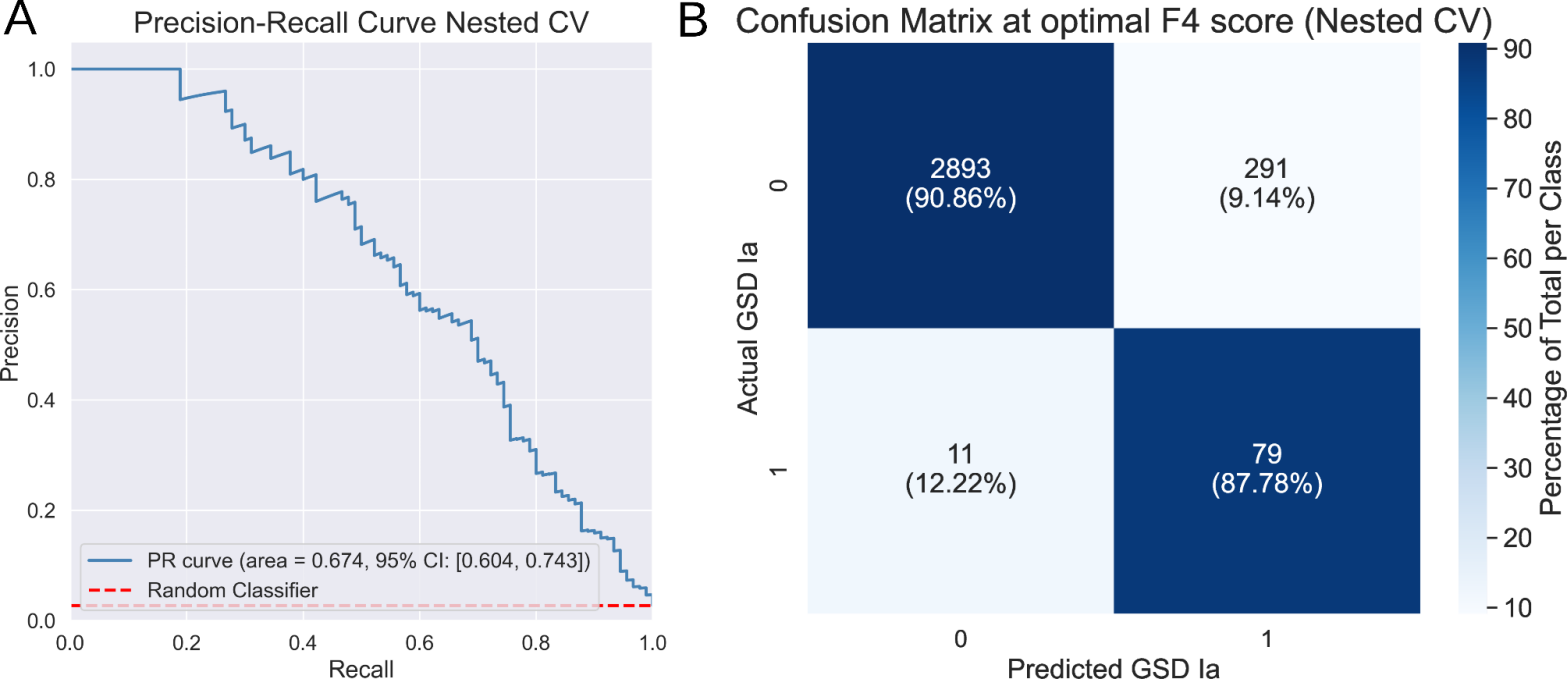
Precision-Recall curve and confusion matrix for Nested CV of the CatBoost classifier. **A** Precision-Recall curve of the best performing CatBoost classifier. Confidence intervals were calculated from the 10 outer folds of the nested CV. **B** Confusion matrix at the cutoff where the F4 score is the highest

The best performing model was a CatBoost model using SVMSMOTE oversampling and 51 features. Figure 2A shows a detailed overview of the performance of this model. It is clear from the confusion matrix (Fig. 2B) that a vast majority of GSD Ia patient samples are correctly identified by this model.

### Evaluation of the model on a held-out test set

**Fig. 3:**
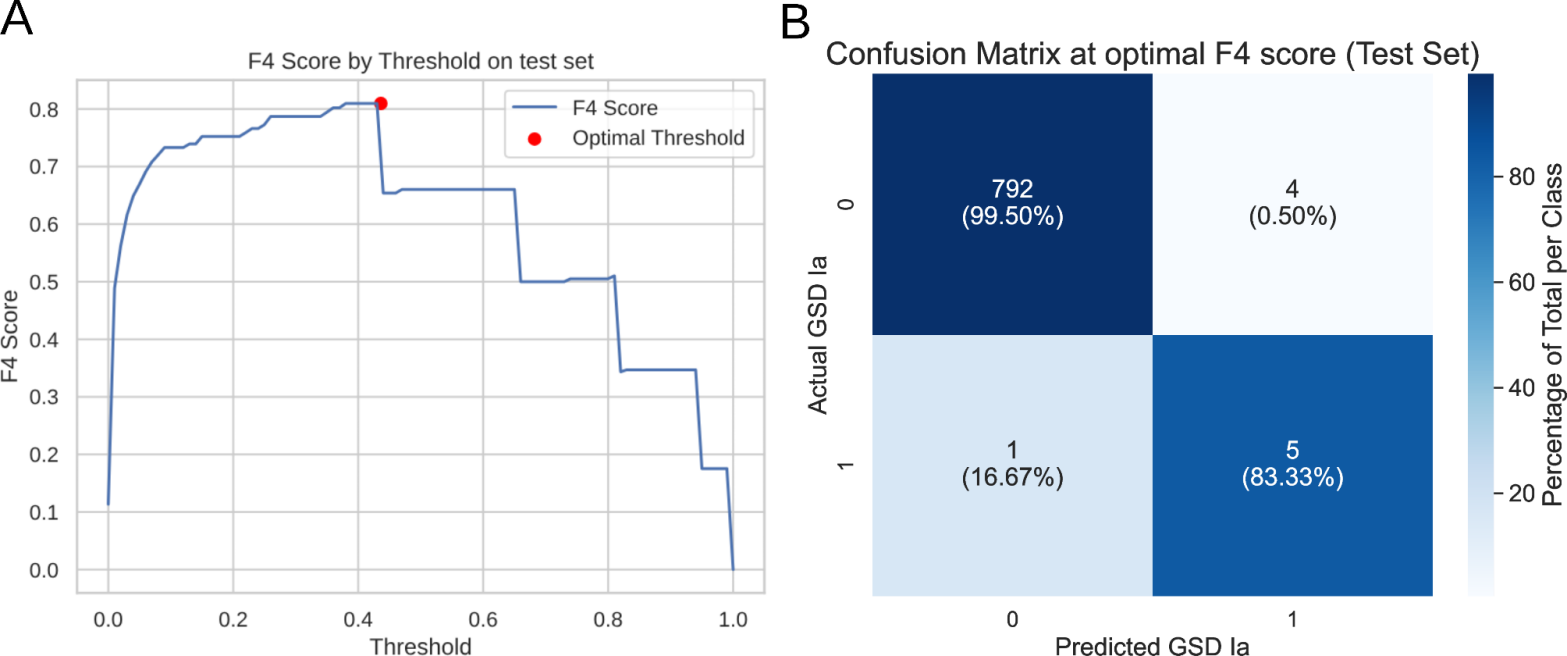
Performance of the CatBoost model on the test set. **A** An overview of F4 scores of the model at different cutoff thresholds, red dot shows optimal cutoff. **B** Confusion matrix of the test set at the threshold with the highest F4 score

To evaluate our final model on out-of-sample data, we trained the model on the full training set using the optimized hyperparameters determined using nested CV and evaluated its performance on the held-out test set. Figure 3A optimal F4 score of the model on the test set and Fig. 3B shows the confusion matrix and. Out of 802 patient samples, we correctly identified five out of six GSD Ia patients with only four false positives using the cutoff optimized for F4 score. This model has an ROC AUC of 0.959 (95% CI: 0.878–1.000), PR AUC of 0.569 (95% CI: 0.168–0.874) and an F4 score of 0.660 (95% CI: 0.330–0.981).

These results show that our model was able to correctly identify the majority of GSD Ia patients in our held-out test set, without generating a significant amount of false positive predictions. The performance of this model on the test set is comparable to the results of nested CV. Moreover, the F4 score is relatively stable across thresholds, indicating a robust model that produces well calibrated predictions. At the optimal threshold, the model performance on the test set yielded a sensitivity of 83.3%, a specificity of 99.5%, a PPV of 55.6% and an NPV of 99.9%.

We looked further into the false positive patients and six GSD Ia patients in the held-out test set. The false positive samples consisted of a patient with methylmalonic aciduria, a one-day old neonate with maternal fatty liver of pregnancy, a patient screened at 3 months of age for developmental delay and hypotonia and a 16-year old patient suffering from muscle weakness. Of the six GSD Ia patients, five exhibited hypertriglyceridemia at the time of sampling (Supplemental Table 2). The one patient that was missed by our model (see Supplemental Fig. 5) was the only GSD Ia patient in the test set with a normal triglyceride concentration (and a severe phenotype based on predicted pathology of the DNA variants) at the time of sampling, suggesting optimal metabolic control.One patient sample was taken prior to diagnosis. In this sample the model correctly identified GSD Ia (see Supplemental Fig. 6).

### Model interpretation

**Fig. 4:**
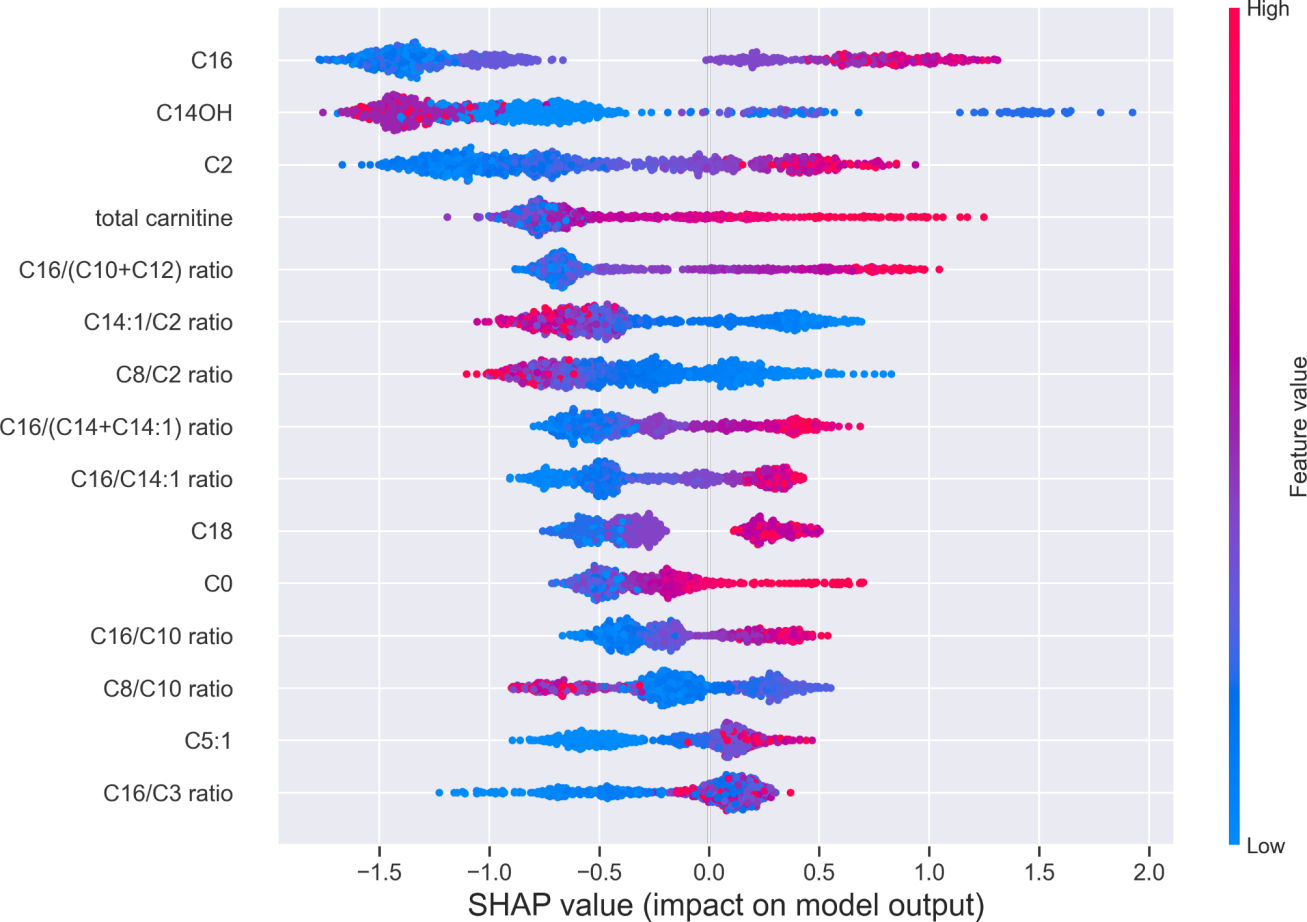
SHAP values of individual data points for the top 15 features in the final CatBoost model when applied to the test set. Red and blue colors represent high and low acylcarnitine concentrations respectively when compared to the mean feature value in the dataset

We used SHAP (SHapley Additive exPlanations) to interpret the model features and their effects on predictions. SHAP is often used to explain how black-box models like gradient-boosted tree algorithms reach their predictions. Figure 4 shows the SHAP summary plot of the top 15 features and how they contribute to model predictions.

A positive SHAP value for a feature means that the feature increases the model prediction for GSD Ia. A negative value decreases the model prediction based on this feature. For example, having a high C16-carnitine (red dots) contributes to a positive prediction of GSD Ia and low C16-carnitine (blue dots) contributes negatively.

From these SHAP values we can clearly see that the most informative features to identify GSD Ia patients are C16-carnitine, total carnitine, free carnitine, acetylcarnitine and other medium and long chain acylcarnitines and their ratios. The model also contains important features that are associated with other IMDs, such as C14:1/C2 ratio (a VLCADD marker), C8/C2 ratio and C8/C10 ratio (both MCADD markers). Interestingly, a model that uses only total carnitine, acetylcarnitine (C2-), C14:1-, C14-, C14OH- and C16-carnitines and some ratios of these was able to perform close to the optimal model shown in Figs. 2, 3 and 4 (see Supplemental Fig. 4). These features were expected to be important as they were the most differentiating in our initial data exploration (Fig. 1).

## Discussion and conclusions

We were able to accurately identify GSD Ia patients based on plasma acylcarnitines profiles using gradient-boosted tree algorithms. In nested, stratified, k-fold CV, our final CatBoost model was able to identify GSD Ia patients with a ROC AUC of 0.955 (95% Ci: 0.934–0.975), PR AUC of 0.674 (95% Ci: 0.604–0.743) and F4-score of 0.488 (95% CI: 0.423–0.552), see Fig. 2. SHAP values confirm that the selected features match acylcarnitine species we expected to be important based on our initial analysis and enzymatic activity studies performed previously [5]. In the held-out test set (802 patients), the model was able to identify five out of six GSD Ia patients with only four false positives (Fig. 3B), associated with a PPV of 55.6% and an NPV of 99.9%. In addition, the single false negative was a GSD Ia patient previously described as having no biochemical abnormalities at the time of sampling [19]. This suggests that the model may be sensitive to the severity of dyslipidemia in patients. This hypothesis is further supported by the very strong positive prediction for the pre-diagnostic GSD Ia patient sample in our test set, which exhibited overt hypertriglyceridemia (see Supplemental Table 2).

Our model’s performance in both nested CV and on the held-out test set was closely comparable, indicating good generalizability of our model. The high negative predictive value (NPV) and sensitivity observed in our study highlight the model’s effectiveness in ruling out GSD Ia in patient samples while also demonstrating strong performance in identifying GSD Ia cases. Importantly, the prevalence of patient samples in our test set aligns with the prevalence observed in our center, ensuring that the metrics are not skewed by an unrepresentative sample distribution. Consequently, we can conclude that our model is robust and reliable for identifying GSD Ia patients.

The features that our model selected can be explained by metabolic changes that occur in individuals with GSD Ia. It is a disorder of both glycogenolysis and gluconeogenesis. The resulting buildup of glucose-6-phosphate leads to secondary shunting to glycolysis and subsequent buildup of acetyl-CoA in the cytoplasm [20]. Elevation of acetyl- (or C2-) carnitine as a direct consequence of this elevation was found in our GSD Ia cohort. High cytoplasmic acetyl-CoA leads to malonyl-CoA production which induces lipogenesis. The main product of lipogenesis is palmitic acid (C16) which can subsequently be elongated to stearic acid (C18). The saturated long chain acylcarnitine species C16 and C18 were indeed significantly elevated in our dataset. Inhibition of CPT I by malonyl-CoA leads to a lower influx of long chain acylcarnitines into the mitochondrial matrix and less substrate for fatty acid oxidation. This fits to the observed decrease in C10-C14 acylcarnitine species. In addition, it was previously shown that VLCAD and MCAD activities are elevated in GSD Ia patients [5] and this could lead to increased flux towards short chain acyl-CoA’s once acyl-CoA’s enter the mitochondrial matrix. This would explain the observed elevations in short chain (C3-C5) acylcarnitines.

Machine learning models have been extensively explored in clinical laboratories [10–14, 21, 22]. Biochemical screening for IMDs generates an enormous amount of data that is very suitable for the development of machine learning models. The dataset we used in this study is a good representation of our clinical diagnostic practice, as it contains samples of patients suspected of an IMD. Most importantly, the dataset contains acylcarnitine profiles of patients with symptoms similar to those of GSD Ia patients, such as hypoglycemia, hyperlactatemia and/or hepatomegaly, due to various causes, including other GSD subtypes.

We used several techniques to deal with the limitations of having a very imbalanced dataset with only a small number of positive samples. Acylcarnitine profiles of all samples of GSD Ia patients, both initial pre-treatment samples (when available) and follow-up measurements, were used in the training dataset for the construction of our models. Initial studies showed a boost in performance using this approach, presumably because multiple samples of a single patient more accurately capture the heterogeneity of possible acylcarnitine profiles in these patients, as we know that acylcarnitine profiles are influenced by decompensation, diet and exercise [23]. In addition, these additional samples can aid in the generation of useful synthetic samples using SVMSMOTE, as the training data is more diverse. Generating synthetic samples using SVMSMOTE improved model performance and proved to be a useful tool to tackle class imbalance in our data, see Table 1.

Our study does have some limitations. The dataset used in this study contains mainly patients who have an established diagnosis and samples are therefore not diagnostic or taken prior to dietary treatment. To assess whether this model could be used in a diagnostic setting, it would need to be prospectively trained and validated on diagnostic samples which we were currently not able to do. Another limitation is that our ML-model only takes acylcarnitines into account, the additional inclusion of other parameters could yield an even stronger model, as GSD Ia patients have hepatomegaly and develop other biochemical abnormalities such as hyperlactatemia, hyperuricemia and hyperlipidemia. In addition, plasma biotinidase activity is known to be significantly elevated in GSD Ia [24], as well as urinary tetraglucoside in a subset of patients [25]. We anticipate that models incorporating these additional parameters will be even more sensitive, potentially capable of detecting patients with mild phenotypes as well. It is currently not fully understood which genetic and/or environmental factors play a role in the heterogeneity between GSD Ia patients, for example in their tendency to develop hypoglycemias, or the risk of developing chronic complications (such as hepatocellular adenomas, and renal complications) [26]. 

Our ML model for detection of GSD Ia patients offers great prospect for inclusion of GSD Ia in newborn screening. GSD Ia is a treatable disease, early diagnosis by NBS will prevent life-threatening hypoglycemia in infancy. Therefore, GSD Ia meets the Wilson & Jungner criteria for population screening [27], and has already been selected for genetic NBS pilot programs [28, 29]. A reliable biomarker in dried blood spots is however lacking. Dried blood spot acylcarnitines are not comparable to those in plasma, the ML model should therefore be built specifically based on DBS acylcarnitine profiles, preferably in combination with biotinidase activities. The application of ML in newborn screening was recently also demonstrated by Jansen et al., who showed that newborn screening for congenital hypothyroidism could be improved by using a machine learning model that incorporated certain amino acids and acylcarnitines [30]. 

This study is one of the first examples of how machine learning can be used in the identification of biochemical profiles of patients with ultra-rare diseases such as GSD Ia. Difficulties inherent to rare disease datasets, such as imbalanced data, small sample size and noise can be adequately addressed using specialized techniques like synthetic oversampling through e.g. SVMSMOTE and the use of multiple samples per patient in the training of models. This work is a first step in addressing the underrepresentation of IMDs amongst studies using ML, and illustrates the opportunities for the application of these techniques, even in ultra-rare diseases.

## Electronic supplementary material

Below is the link to the electronic supplementary material.

Supplementary Material 1

## Data Availability

The datasets used and/or analysed during the current study are available from the corresponding author on reasonable request. The Python script used to build the machine learning models in this study is freely available on GitHub [17].

## Abbreviations

GSD Ia: Glycogen storage disease type Ia
ML: Machine learning
Nested CV: Nested cross-validation
ROC AUC: Area under the receiver operating characteristic
PR AUC: Are under the precision-recall curve
G6Pase: Glucose-6-phosphatase
CPT I: Carnitine palmitoyltransferase I
MCADD: Medium chain acyl-CoA dehydrogenase deficiency
VLCADD: Very long chain acyl-CoA dehydrogenase deficiency
IMD: Inherited metabolic disease; KNN, k-nearest neighbors
RF: Random forests
XGBoost: Extreme gradient boosting
SVMSMOTE: Support vector machine synthetic minority over-sampling technique
SHAP: Shapley additive explanations
PPV: Positive predictive value
NPV: Negative predictive value
