# Supplementary material for: A machine learning model accurately identifies glycogen storage disease Ia patients based on plasma acylcarnitine profiles
Source: Orphanet J Rare Dis. 2025 Jan 9;20:15. doi: 10.1186/s13023-025-03537-2 (PMC11721056; doi:10.1186/s13023-025-03537-2)
Supplement: Supplementary file 1 — Supplementary Material 1 [file 13023_2025_3537_MOESM1_ESM.docx]

**Supplemental Material**

**List of abbreviations and terms**

**Bagging:** Training multiple models independently and averaging their predictions to achieve a final prediction.

**Boosting:** Iteratively training multiple models, adding weight to samples misclassified by the previous model. The predictions of these models are weighted according to their accuracies leading to a strong classifier.

**F4 score:** Extension of the F1 score. In the F1 score, precision and recall are balanced equally. The F4 score assigns four times more weight to recall than to precision. It can be applied when false negatives are considered more costly than false positives. The formula is given below:

$$F4 score=\left( 1+4^{2} \right)\times\frac{Precision \times Recall}{\left( 4^{2}\times Precision \right)+Recall}$$

**KNN imputation:** Imputation of feature values using k-nearest neighbor algorithm. Finds the k most similar datapoints (nearest neighbors) and imputes the missing value based on the average of this parameter in the k-nearest neighbors.

**SVMSMOTE:** An algorithm that uses Support Vector Machines to generate synthetic minority class samples at the borderline areas where majority and minority samples overlap. This aims to provide more informative synthetic samples compared to regular SMOTE.

**Undersampling:** Removing majority class samples from the train set to obtain a more equal sample distribution.

**Supplemental Table 1** Description of the IMD's of patients in the full dataset.

| Inherited Metabolic Disease | Number of patients (total = 3958) |
| --- | --- |
| None | 3523 |
| PKU (phenylketonuria) | 100 |
| MCADD (medium chain acyl-Coa dehydrogenase deficiency) | 85 |
| Other fatty acid oxidation disorders | 37 |
| Other GSD’s (glycogen storage diseases) | 34 |
| GSD Ia (glycogen storage disease Ia) | 31 |
| Mitochondrial disorders | 28 |
| MMA/PA (methylmalonic acidemia / propionic acidemia) | 25 |
| Lysosomal storage disorders | 13 |
| Urea cycle disorders | 13 |
| Cystinosis | 9 |
| IVA (isovaleric acidemia) | 7 |
| Biotinidase deficiency | 6 |
| GA-I (glutaric acidemia type I) | 5 |
| Peroxisomal biogenesis disorder | 5 |
| GLUT1 deficiency | 4 |
| LPI (lysinuric protein intolerance) | 4 |
| Other | 29 |

**Supplemental Table 2** Characteristics of GSD Ia patients included in the dataset. Triglyceride and uric acid concentrations are those of the first samples of these patients, which are the ones used in validation sets during nested cross-validation.

| True positive (TP) or False negative (FN) | Sex | Age at clinical presentation | Triglyceride (mmol/L) at time  of sampling | Uric acid  (mmol/L) at time of sampling | Homozyogous for predicted severe variants | Reference |
| --- | --- | --- | --- | --- | --- | --- |
| Test set  Found TP | F | 1 mo | 3.09 | 0.39 | No | Case 4 in PMID: 28397058  Case 12 in PMID: 35811762 |
| Test set  Found TP | M | 11 mo | 2.08 | 0.29 | No | Case 43 in PMID: 35811762 |
| Test set  Found TP | F | 3 mo | 5.90 | 0.61 | Yes | Case 14 in PMID: 35811762 |
| Test set  Found TP | M | 21 mo | 3.31 | 0.29 | No | Case 2 in PMID: 28397058  Case 30/31 in PMID: 35811762 |
| Test set  Found FN  Supplemental Figure 5 | F | 22 mo | 1.79 | 0.29 | Yes | Case D in PMID: 28397058  Case 44 in PMID: 35811762 |
| Test set  Found TP  Sample prior to diagnosis and treatment.  Supplemental Figure 6 | M | 9 mo | 11.29 | 0.26 | Yes | Not published |
| Train sample | F | 2 mo | 7.10 | 0.39 | Yes | Case 8 in PMID: 35811762 |
| Train sample | M | 10 mo | 8.78 | 0.25 | Yes | Case 2 in PMID: 35811762 |
| Train sample | F | 5 mo | 3.87 | 0.24 | Yes | Case 16 in PMID: 35811762 |
| Train sample | F | unknown | 3.33 | na | unknown | Not published |
| Train sample | M | 3 mo | 7.42 | 0.36 | Yes | Case 14 in PMID: 35811762 |
| Train sample | M | 3 mo | 6.84 | 0.28 | unknown | Not published |
| Train sample | M | 10 mo | 5.92 | 0.28 | No | Case 33 in PMID: 35811762 |
| Train sample | M | 225 mo | 2.64 | 0.33 | Yes | Case 28 in PMID: 35811762 |
| Train sample | F | unknown | 3.19 | 0.25 | unknown | Not published |
| Train sample | F | 13 mo | 1.16 | 0.15 | Yes | Case 27 in PMID: 35811762 |
| Train sample | M | 2 mo | 5.78 | 0.31 | No | Case 20 in PMID: 35811762 |
| Train sample | M | unknown | 1.27 | 0.32 | unknown | Not published |
| Train sample | F | 13 | 3.84 | 0.28 | Yes | Case 6 in PMID: 35811762 |
| Train sample | M | unknown | 10.22 | 0.32 | Yes | Case 42 in PMID: 35811762 |
| Train sample | M | 1 mo | 4.27 | 0.44 | No | Case 5 in PMID: 35811762 |
| Train sample | F | unknown | 5.84 | unknown | unknown | Not published |
| Train sample | F | 0 mo | 6.45 | 0.16 | Yes | Case 9 in PMID: 35811762 |
| Train sample | M | unknown | 4.14 | 0.40 | unknown | Not published |
| Train sample | M | 31 mo | 4.03 | 0.29 | No | Case 36 in PMID: 35811762 |
| Train sample | F | 5 mo | 24.39 | 0.36 | Yes | Case 3 in PMID: 35811762 |
| Train sample | F | 12 mo | 5.04 | 0.40 | unknown | Not published |
| Train sample | F | 10 mo | 15.22 | 0.45 | Yes | Case 40 in PMID: 35811762 |
| Train sample | M | 0 mo | 5.37 | 0.31 | unknown | Not published |
| Train sample | M | 1 mo | 105.66 | 0.20 | No | Case 17 in PMID: 35811762 |
| Train sample | F | 11 mo | 6.61 | 0.42 | Yes | Case 1 in PMID: 35811762 |

**PMID 28397058:** Peeks F, Steunenberg TAH, de Boer F, Rubio-Gozalbo ME, Williams M, Burghard R, et al. Clinical and biochemical heterogeneity between patients with glycogen storage disease type IA: the added value of CUSUM for metabolic control. J Inherit Metab Dis 2017;40:695.

**PMID 35811762:** Haring MPD, Peeks F, Oosterveer MH, Brouwers MCGJ, Hollak CEM, Janssen MCH, et al. High childhood serum triglyceride concentrations associate with hepatocellular adenoma development in patients with glycogen storage disease type Ia. JHEP Rep 2022;4(8):100512.


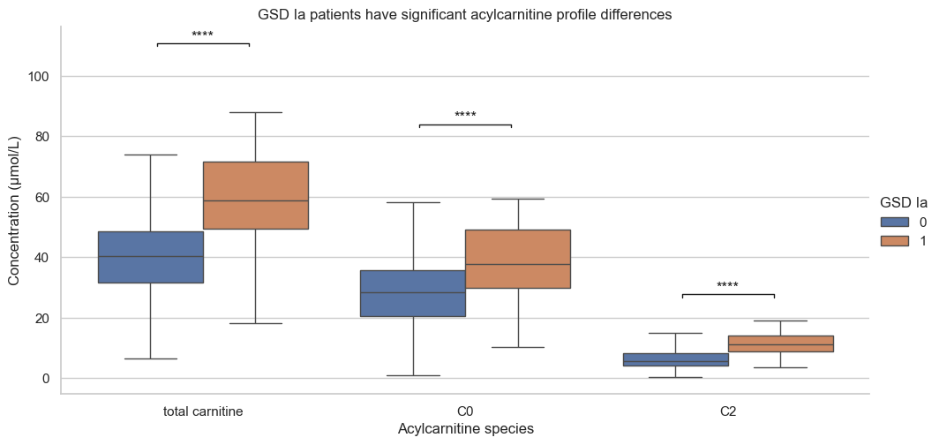


**Supplemental Figure 1** Concentrations of total carnitine, free carnitine (C0) and acetylcarnitine (C2) in both groups.


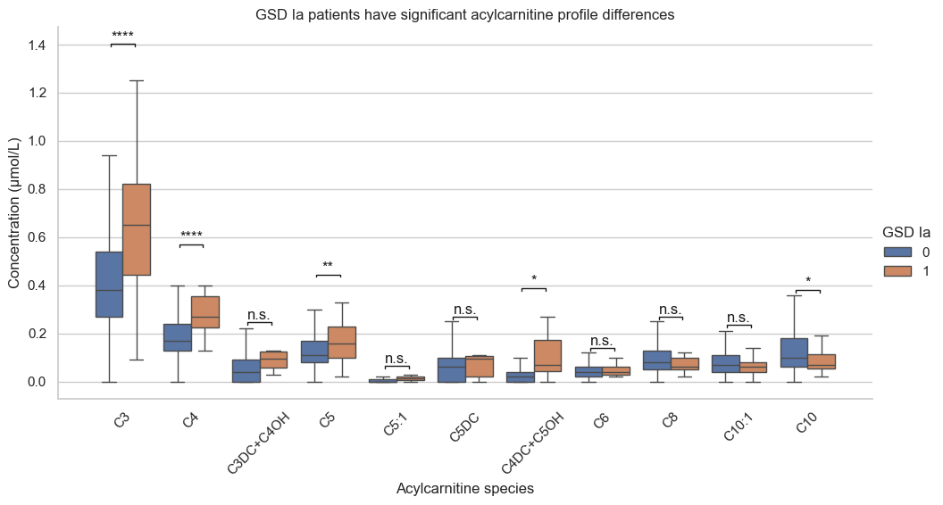


**Supplemental Figure 2** Concentrations of short and medium chain acylcarnitines in both groups.


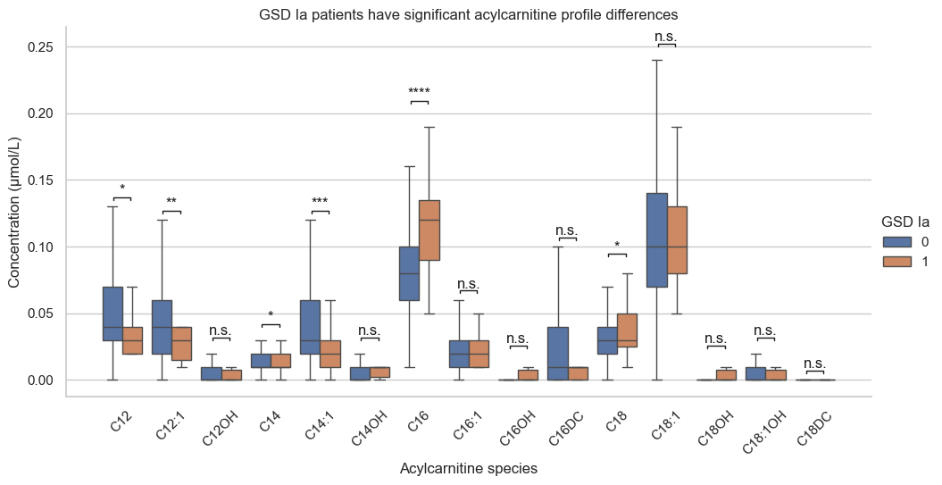


**Supplemental Figure 3** Concentrations of medium and long chain acylcarnitines in both groups.


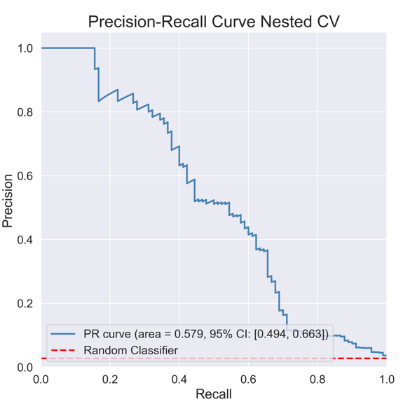

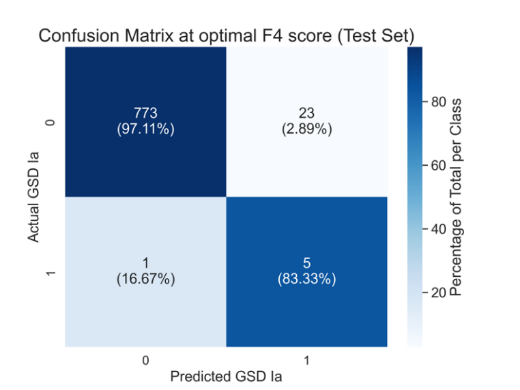

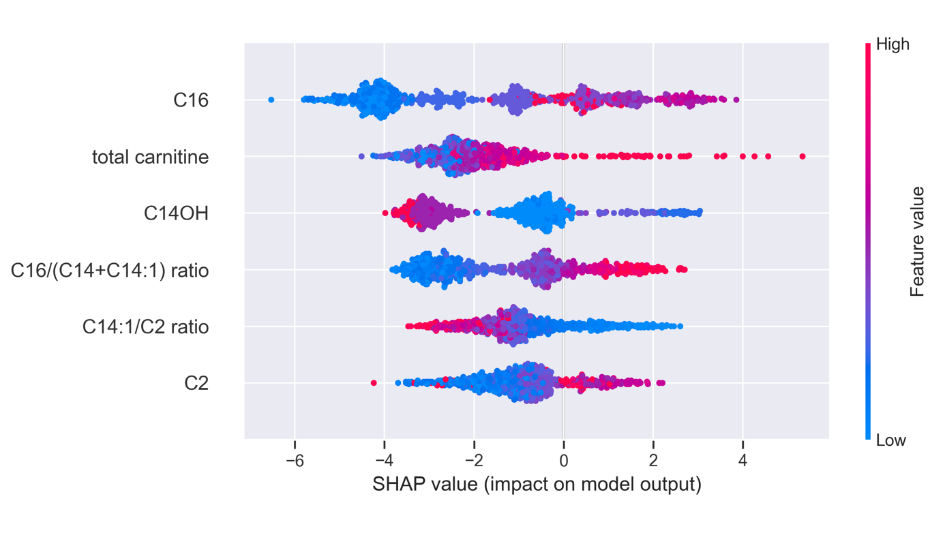


**Supplemental Figure 4** Performance of a model where only the top 10% of features by feature importance are selected (6 features). **A** ROC curve and PR curve of nested cross-validation of this model. **B** Confusion matrix of the test set of the model at the cutoff with optimal F4 score. **C** SHAP values of the 6 selected features of this model.


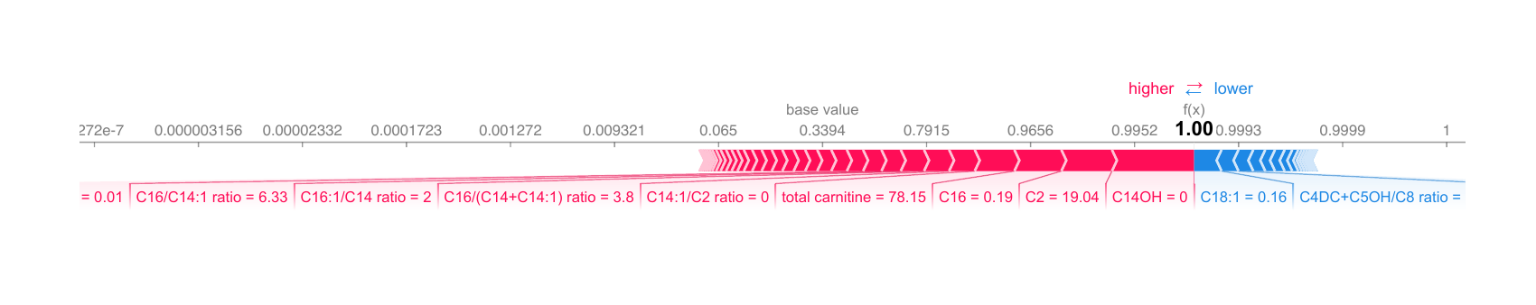

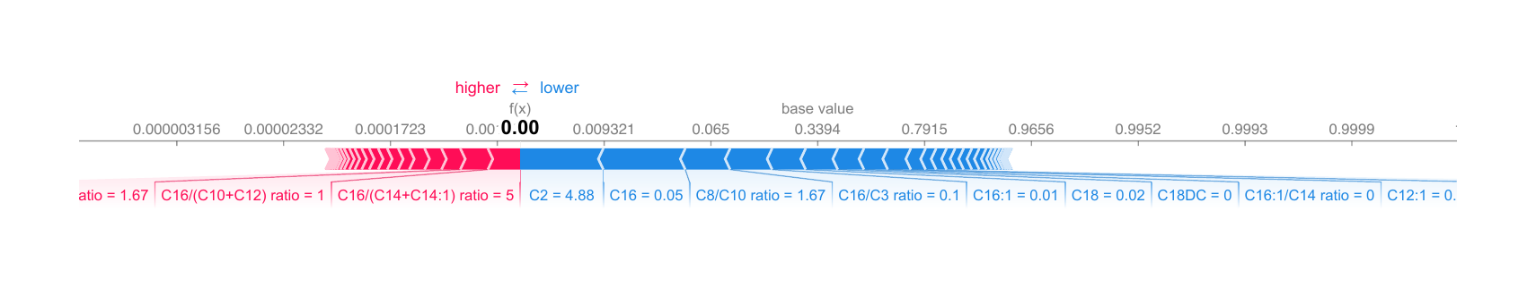


**Supplemental Figure 6** SHAP force plot of a GSD Ia patient sample taken untreated and prior to diagnosis. The most important features for GSD Ia prediction are all present in this patient smaple, leading to a strong prediction of GSD Ia.

**Supplemental Figure 5** SHAP force plot of the GSD Ia patient that was not identified by the ML model. This patient showed no elevated total carnitine, free carnitine, acetylcarnitine or C16 carnitine.
